# *CMTM8* variants influence BNT162b2 COVID-19 vaccination response by regulating granulocytic/polymorphonuclear myeloid-derived suppressor cell activity

**DOI:** 10.3389/fimmu.2026.1717058

**Published:** 2026-01-23

**Authors:** Alessandro Testori, Antonella Mulas, Mara Marongiu, Valeria Orrù, Monia Lobina, Maria Grazia Piras, Erika Lutzu, Nicolò Curreli, Cristina Politi, Marco Mobrici, Giorgio Iervasi, Daniela Corda, Mario De Felice, Alessandra Testa, Marcella Devoto, Maristella Steri, Edoardo Fiorillo, the SerGenCovid-19 Investigators

**Affiliations:** 1 Istituto di Ricerca Genetica e Biomedica (IRGB) - Consiglio Nazionale delle Ricerche (CNR), Cagliari, Italy; 2 Istituto di Ricerca Genetica e Biomedica (IRGB) - Consiglio Nazionale delle Ricerche (CNR), Lanusei, Italy; 3 Istituto di Fisiologia Clinica (IFC) - Consiglio Nazionale delle Ricerche (CNR), Reggio Calabria, Italy; 4 Istituto di Fisiologia Clinica (IFC) - Consiglio Nazionale delle Ricerche (CNR), Pisa, Italy; 5 Istituto degli Endotipi in Oncologia, Metabolismo e Immunologia “G. Salvatore” (IEOMI), - Consiglio Nazionale delle Ricerche (CNR), Napoli, Italy; 6 Dipartimento di Medicina Traslazionale e di Precisione, Sapienza Università di Roma, Rome, Italy

**Keywords:** BNT162b2, CMTM8, GWAS, HLA, MDSC (myeloid-derived suppressor cells)

## Abstract

**Background:**

The immunoglobulin level following vaccination against SARS-CoV-2 results from a multifaceted immunological process involving cells and molecules that determine its efficacy and protect us against severe infection outcomes. The observed heterogeneity in the immune response to vaccination is partly attributable to host genetic factors; however, this genetic contribution has only been partially explored.

**Methods:**

To elucidate the mechanisms underlying the antibody response elicited by the Pfizer-BioNTech vaccine, we conducted a genome-wide association study on anti-spike immunoglobulin G levels measured in 1,968 Italian individuals who received two doses of the vaccine, selected from a larger cohort of 7,169 volunteers characterized for 8 million genetic variants. Sex, age, body mass index, smoking habit, and time elapsed between vaccine administration and blood draw were accounted as covariates in the linear regression model.

**Results:**

We identified a novel signal of association on chromosome 3 in the intronic region of the *CMTM8* gene and confirmed one previously identified at the *HLA* locus close to the *HLA-B* gene. The lead SNP in the *CMTM8* gene, rs7643677 (*p*-value = 2.095×10^-8^), is associated with anti-S IgG levels and with the expression level of CD66b on granulocytic/polymorphonuclear myeloid-derived suppressor cells.

**Conclusions:**

These findings support a role for *CMTM8* in regulating the suppressive activity of specific immune cells, and suggest a potential interplay among genetic, humoral, and cellular mechanisms underlying the immune response to SARS-CoV-2 vaccination.

## 1 Background

The emergence of the SARS-CoV-2 pandemic prompted an unprecedented research effort toward the development of novel vaccination strategies, leading to the identification of effective compounds against this virus in a remarkably short time (1, 2). Indeed, several mRNA vaccines were developed, and the technology has proven to be extremely safe and effective. The Pfizer-BioNTech COVID-19 mRNA vaccine received full approval in the European Union, the United Kingdom, and the United States by the end of 2020 (3). It consists of a synthetic nucleoside-modified mRNA molecule (BNT162b2) inserted into lipid nanoparticles (LNPs), specifically ALC-0315 (4, 5). Following injection, the LNPs deliver BNT162b2 to host cells, where the mRNA is translated into an immunogenic antigen by the host’s translational machinery. This antigen is then presented via the major histocompatibility complex (MHC) class I pathway, which involves the *HLA-A*, *-B*, and *-C* genes and displays peptides derived from within the cell (6, 7).

The exact immunological mechanisms underlying the efficacy of the BNT162b2 vaccine remain poorly understood (8). Following widespread COVID-19 immunization campaigns using this and other vaccines, genetic association studies have been conducted to investigate whether an individual’s genetic background may influence vaccination efficacy (9–13). The *HLA* region has been associated with anti-spike immunoglobulin G (anti-S IgG) titer following vaccination, as well as with disease severity and susceptibility (14). Additional loci have reached suggestive levels of significance in genetic association analyses (*p*-value < 10^-6^), although considerable variability has been observed in results across different studies (11, 13).

**Figure 1:**
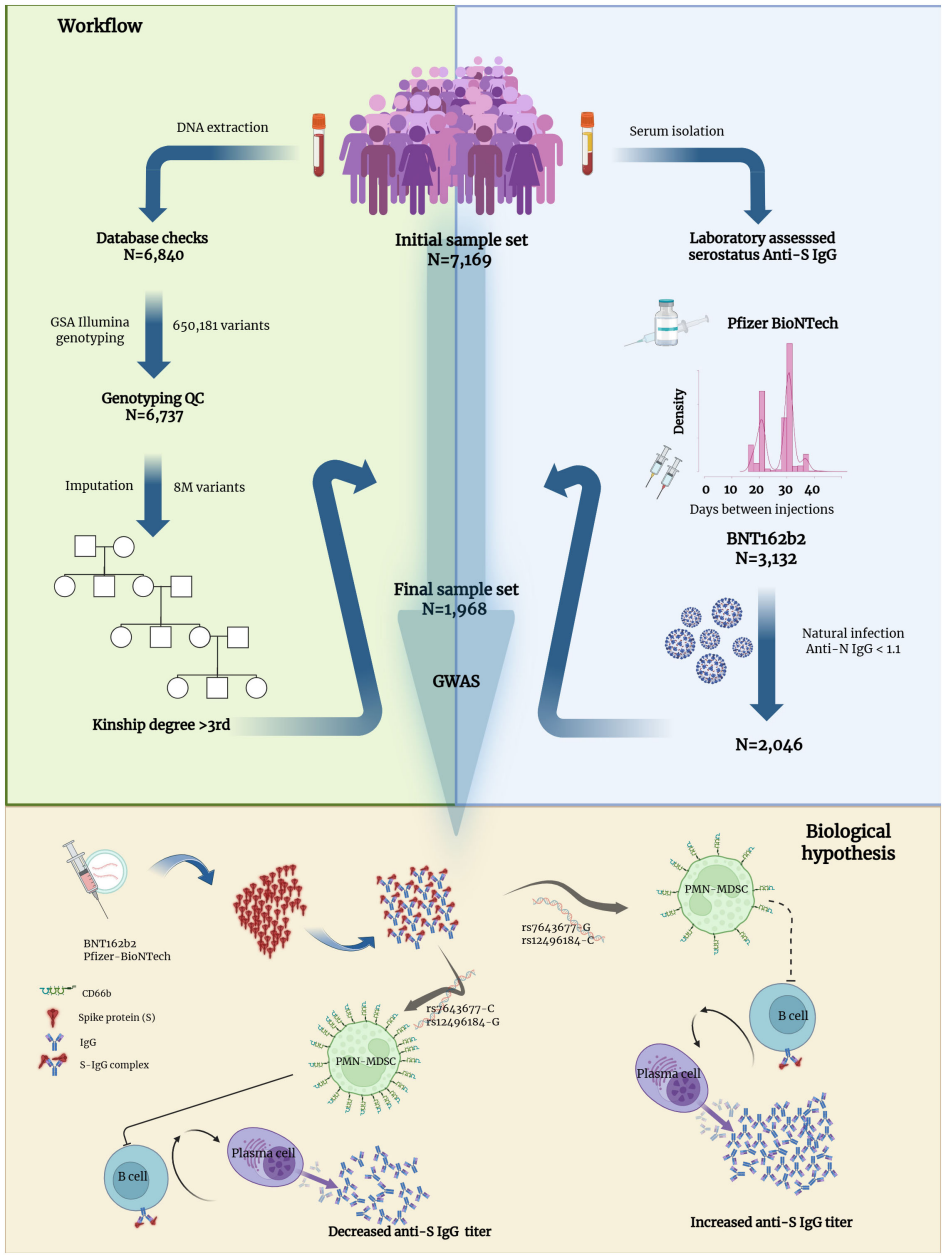
Flow chart describing phenotype definition, study sample selection criteria, and biological hypothesis. The study involved 7,169 individuals who participated in the SerGenCovid-19 study. The light-blue panel of the workflow illustrates the serological data processing. The primary phenotype analyzed was the level of SARS-CoV-2 spike protein antibody (S antibody) following BNT162b2 vaccine, measured in Binding Antibody Units per milliliter (BAU/ml). Samples from individuals vaccinated with other vaccine types were excluded. Eligible samples included participants with consistent information across longitudinal data and a time interval of no more than 40 days between the first and second dose of vaccine. Individuals showing evidence of natural infection, detected by positivity for the SARS-CoV-2 nucleocapsid protein (N), were excluded. The light-green panel illustrates the sample processing workflow, from blood collection to genetic analysis. DNA was automatically extracted from frozen samples and genotyped in-house. Standard quality control filters were applied, resulting in a dataset of over 8 million variants (both genotyped and imputed) that were used for the GWAS. The bottom light-orange panel illustrates the proposed biological mechanism underlying the early stages of the immunological response to vaccination and schematically depicts the proposed role of the GWAS-identified variants. The solid line between PMN-MDSC and B cell indicates stronger inhibition compared to the dashed line.

Here, we conducted a Genome Wide Association Study (GWAS) on anti-S IgG levels following Pfizer-BioNTech COVID-19 vaccination in 1,968 participants from the SerGenCovid-19 initiative, a cohort study comprising 7,169 genetically characterized individuals from the general Italian population. We analyzed over 8 million genetic variants in all participants who joined the study between May 2021 and February 2022 and had received two doses of the vaccine (**Figure 1**). We confirmed one previously reported signal in the *HLA* region and identified a variant in the *CKLF Like MARVEL Transmembrane Domain Containing 8* (*CMTM8)* gene. *CMTM8* belongs to the chemokine-like factor gene superfamily and acts as a tumor suppressor, immune chemotactic activator, and regulates cell proliferation, differentiation, and apoptosis (15, 16). Interestingly, the strongest expression has been demonstrated in the pancreas, a critical SARS-CoV-2 target, as SARS-CoV-2 infiltration of exocrine and endocrine pancreatic cells both *in vitro* and *in vivo*, has been linked to pancreatic impairment (17). The *CMTM8* variant is associated with the activation level of granulocytic/polymorphonuclear myeloid-derived suppressor cells (PMN-MDSCs), a dynamic population of myeloid origin, featuring the capacity to suppress the adaptive immune response (18). In general, expansion of PMN-MDSCs has been described as positively correlated with a variety of pathological conditions, including sepsis and acute inflammatory responses (19, 20), recently considered as a predictor of COVID-19 disease severity (21–23). A smoldering body of evidence supports a role for MDSCs in non-pathological settings such as vaccination, where they can limit vaccine efficacy (24, 25). Our findings contributed to elucidating the immunological mechanisms underlying the efficacy of the BNT162b2 vaccine.

## 2 Materials and methods

### 2.1 Study cohort and data collection

The samples used in this study are part of the SerGenCovid-19 project (https://sergencovid19.d4science.org), an initiative of the Italian National Research Council (Consiglio Nazionale delle Ricerche, CNR) aimed at identifying the genetic and serological factors involved in response to vaccination against COVID-19 and susceptibility to SARS-CoV-2 infection. Before blood sampling, participants in the SerGenCovid-19 project were asked to provide health, anamnestic, and lifestyle information through a secure, access-restricted online questionnaire. Blood samples were collected over a period of approximately nine months, resulting in the creation of a biobank representative of the Italian population. The SerGenCovid-19 project successfully collected data from 7,169 participants across Italy.

All participants provided written informed consent to the study protocol (approval n. 273 of the experimentation registry - 03/Feb/2021, Ethics Committee of the National Institute for Infectious Diseases Lazzaro Spallanzani I.R.C.C.S.).

Sample collection was conducted between May 2021 and February 2022. The personal and clinical information collected included age, sex, smoking status, dates and types of vaccination, and date of blood draw. Peripheral blood samples were obtained to extract genomic DNA and isolate serum. Antibody titers against the SARS-CoV-2 spike protein (anti-S) and nucleocapsid protein (anti-N) were measured in serum. Anti-S IgG levels were assessed using the LIAISON SARS-CoV-2 TrimericS IgG assay (DiaSorin), and overall anti-N antibodies were evaluated with the Elecsys Anti-SARS-CoV-2 assay (Roche).

Measurements were taken at a median time of 87 days after administration of the second dose (range: 1–309 days; mean: 103 days; interquartile range: 114 days). To accurately assess vaccine response, 1,086 samples showing serologic evidence of prior SARS-CoV-2 infection (anti-nucleocapsid antibody levels > 1.1 UM ICO) were excluded. The remaining sample set was further restricted based on the type of vaccine administered, retaining only participants who received two doses of the BNT162b2 (Pfizer-BioNTech) vaccine within a 40–day interval and who passed data quality control filters (see below; n=2,046). Related individuals up to the third degree of kinship were excluded, resulting in a final sample size of 1,968 individuals.

### 2.2 Genome-wide genotyping, imputation, and data quality control

Blood samples were collected in EDTA tubes, and genomic DNA was extracted using the QIAcube QIAamp DNA Mini Kit following an automated protocol. Genotyping was performed using the Infinium™ Global Screening Array-24 v3.0 BeadChip (Illumina, San Diego, CA, USA), which assays approximately 650K markers across the human genome.

The GenomeStudio^®^ 2.0 Data Analysis Software was used to normalize, cluster, and call genotypes with a standard call rate of > = 98%. Samples with a call rate below 98% were excluded from further analysis.

Quality control (QC) of genotype data was then performed using PLINK v1.9 (26). Only samples with a missing rate < 2% were retained. In addition, individuals were included only if they showed concordance between reported and genetically inferred sex, as well as no excess heterozygosity (i.e., within the mean +/-3 standard deviations).

Markers were filtered by only including variants with minor allele frequency (MAF) higher than 0.01, and with a Hardy-Weinberg equilibrium test *p*-value > 1 × 10^−5^.

To generate a high-density genetic map, genotype imputation was performed on quality-controlled genotypes using Minimac via the TOPMed Imputation Server (27) and the TOPMed-r2 reference panel, which comprises haplotypes from approximately 93,000 deeply sequenced individuals of diverse ancestry. First, genotype data were phased to the reference panel with Eagle v2.4 (28). SNPs excluded during pre-imputation QC because they did not match the reference panel were subsequently switched or flipped as appropriate. After imputation, only variants with high imputation quality (r^2^ info score >= 0.3 for MAF >= 0.05, or r^2^ info score >= 0.6 for 0.01=<MAF < 0.05) were included, yielding a total of 8,080,429 variants (7,557,722 SNPs and 522,707 indels) suitable for downstream analyses.

### 2.3 Statistical analysis

The null hypothesis of normality of the anti-S IgG level distribution was tested using the Kolmogorov-Smirnov test and was rejected (*p*-value < 0.05). The quantitative variable was thus normalized using an inverse normal transformation, as in the work by Yang and colleagues (29).

A linear regression analysis was performed with the *lm()* function of R to evaluate the association between anti-S IgG levels and genotypes, using sex, age, body mass index (BMI), smoking status (yes or no), and time elapsed between vaccination and blood collection as covariates. These independent variables (amongst several others that accounted for biometrical parameters, as well as comorbidities and treatments) were included in the model as they significantly affected IgG levels (model adjusted R^2^ = 0.43). GWAS was performed on anti-S IgG levels using a linear regression model in PLINK (*–linea*r function).

Genomic control was assessed through the *lambda* statistic using PLINK (–*adjust* option). Manhattan and QQ plots were generated using the *qqman* library in R (30). Regional association plots were created using the *locuszoomr* library in R (31).

Conditional association analyses were performed at the two most significant GWAS signals on chromosomes 3 and 6 using an additive linear model. At each step, the lead SNP was included as a covariate to the model adjusted for sex, age, BMI, smoking status, and time elapsed between the second vaccine dose and blood draw using PLINK’s –*cond* function. Replication of the GWAS findings was evaluated by comparing our results with previously published studies on similar phenotypes (10, 12) and in COVID-19-related GWAS (https://www.covid19hg.org/) (14).

Genetic colocalization analysis was performed to test whether our phenotype shared causal genetic variants with immune traits investigated in other studies using the *coloc* tool (32). Specifically, we searched for the presence of our genome-wide significant lead variants, rs2596436 and rs7643677, in the summary statistics of the Sardinian population immune profile database (33), which includes 731 quantitative immune traits comprising 118 cell counts, 192 cell proportions, 389 expression levels of surface antigens median fluorescence intensities (MFI), and 32 morphological parameters from 3,757 individuals. For both of the above variants, we selected the immunophenotype with the most significant association *p*-value, and colocalization analysis was then performed in a genomic region spanning +/- 100 kb from the associated variant.

If our lead SNPs were not present in the datasets used for replication or colocalization analyses (12, 33), the variant/s in strongest linkage disequilibrium (LD, r^2^≥0.8) with the lead variants were used instead.

For any given SNP pair, linkage disequilibrium and in-phase alleles (i.e., alleles co-inherited on the same haplotype) were obtained using PLINK *(–ld* option).

Fine mapping was applied to GWAS results to identify potential causal variant(s) and candidate gene(s).

Genetic variants were annotated using the Ensembl Variant Effect Predictor (https://www.ensembl.org/Tools/VEP) to assess their potential functional consequences and overlap with known genomic features. Credible sets were defined using the *finemap.abf* function of the *coloc* package (32). A posterior probability of being causal was assigned to each variant, and a *p*-value was reported; variants were then sorted in descending order of probability and included in the credible set until the cumulative sum reached 95%. eQTL search for the lead genome-wide significant variants was performed using the information reported in the Linda database (http://linda.irgb.cnr.it).

## 3 Results

We analyzed anti-S IgG levels in a selected sample set of 1,968 individuals who received two doses of the Pfizer-BioNTech vaccine and did not show evidence of prior infection, ascertained by excluding individuals with N-protein seropositive results. Anti-S IgG levels had a median of 802 BAU/ml (range: 4.81-39,800 BAU/ml; mean: 1,351.83 BAU/ml; IQR: 1,267 BAU/ml). We observed a decrease in anti-S IgG titer proportional to the time elapsed between the two subsequent injections (**Supplementary Figure S1**). We also found that IgG levels were inversely correlated with age (**Supplementary Figure S2A**) and positively correlated with BMI (**Supplementary Figure S2B**). Moreover, in line with previous reports (34) we found a higher IgG titer in non-smokers compared to smokers (**Supplementary Figure S2C**). In contrast to another study (12), we observed significantly higher levels in females compared to males (**Supplementary Figure S2D**, **Supplementary Table S1**).

To identify genetic polymorphisms associated with variation in anti-S IgG levels following vaccination against SARS-CoV-2, we performed a GWAS, testing more than 8 million markers. Sex, age, BMI, smoking status, and time between the second vaccine dose and blood draw were included as covariates in the linear regression model (**Supplementary Table S1**).

**Figure 2:**
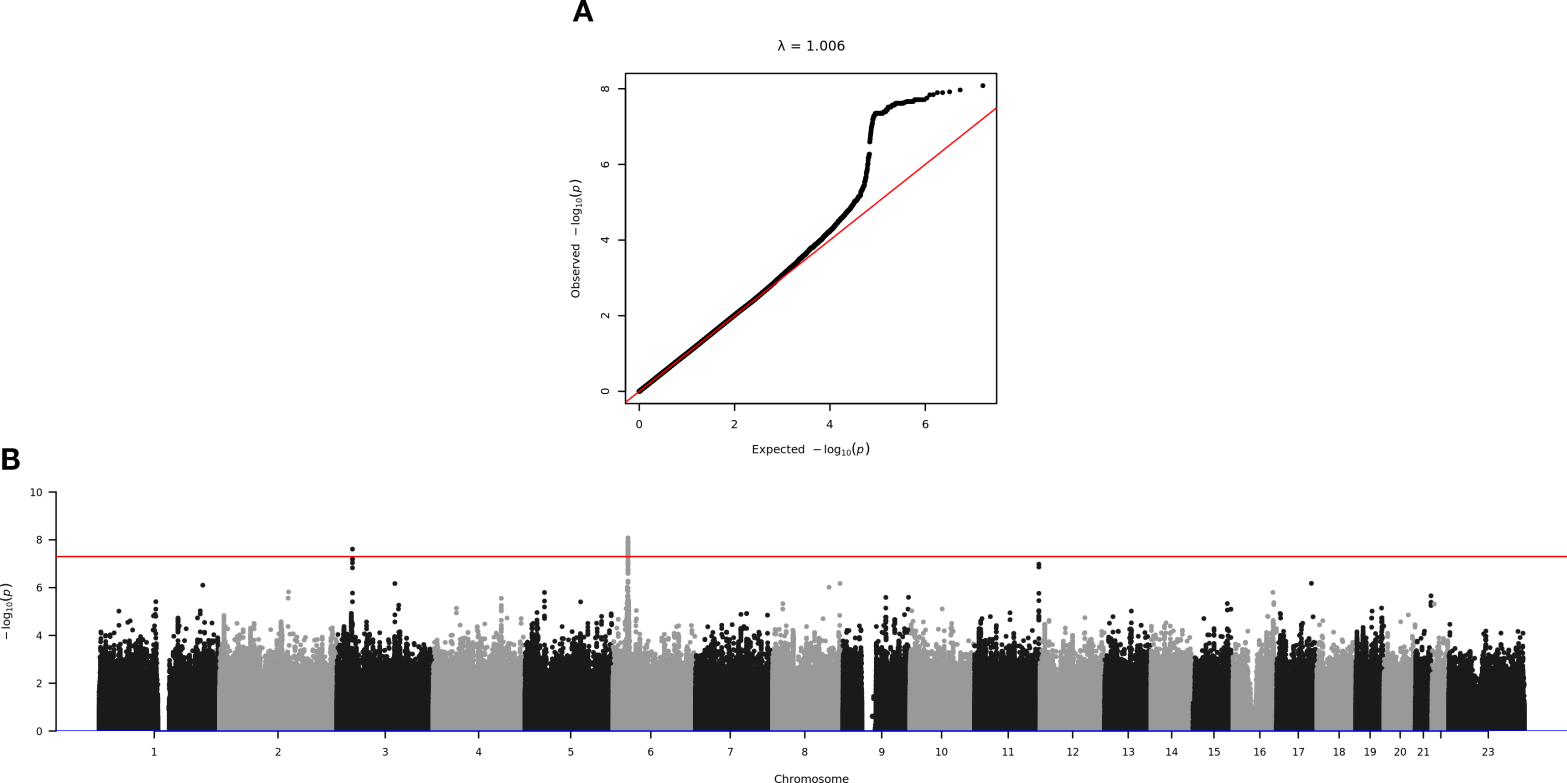
**(A)** Q-Q plot of observed and expected *p*-values. The genomic inflation factor λ is equal to 1.006. **(B)** Manhattan plot of the GWAS on the inverse normalized anti-spike IgG values against >8 million variants. SNPs are plotted on the x-axis according to their genomic position (hg38) and *p*-values (−log_10_(p)) for their association with IgG levels on the y-axis. The horizontal red line represents the standard genome-wide significance threshold (*p*-value = 5 × 10^−8^).

**Figure 3:**
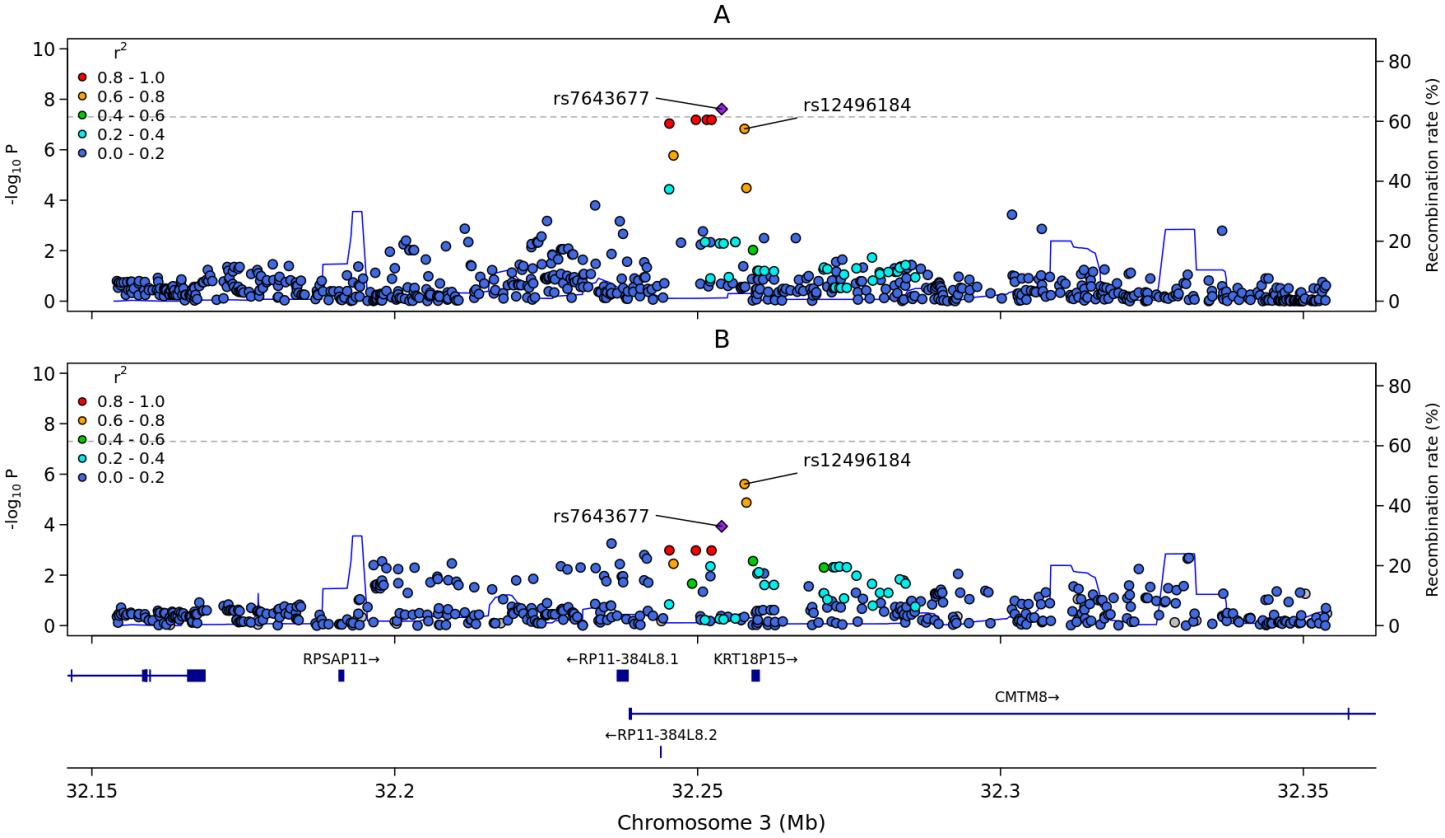
Regional association plots of the chromosome 3 locus identified in the GWAS. Plots span a region encompassing ± 100 Kb from the lead variant rs7643677. SNPs are plotted on the x-axis according to their position on chromosome 3, and *p*-values [−log_10_(p)] for their association with the traits are plotted on the y-axis. Variants are colored according to their LD (r^2^) with the lead SNP (purple diamond). **(A)** Regional plot from the GWAS on the anti-S IgG. **(B)** Regional plot for the immune trait (CD66b on Gr MDSC).

After confirming the absence of population stratification in our data (λ = 1.006, **Figure 2A**), we identified a novel genome-wide significant signal of association in the intronic region of the *CMTM8* gene on chromosome 3 (**Figures 2B**, **3A**; **Supplementary Table S2**).

The C allele of the lead variant in *CMTM8*, rs7643677, was associated with reduced anti-S IgG levels (effect=-0.099 standard deviation units, *p*-value = 2.095×10^-8^). Conditional analysis revealed no additional signal significantly associated with anti-S IgG levels in this region, indicating that the signal led by rs7643677 fully accounted for the observed association (**Supplementary Table S3**). Fine-mapping analysis assigned a probability of 38% to rs7643677 being the causal variant (**Supplementary Table S4**).

To investigate the biological mechanisms underlying the genetic associations with anti-S IgG levels, we assessed whether the lead SNP had been reported as an expression Quantitative Trait Locus (eQTL) for genes in the region. We found that rs7643677 was in high LD (r^2^ = 0.98) with SNP rs6550109, a known eQTL for *CMTM8* in whole blood (*p*-value = 6.24×10^-130^), where the rs6550109-T allele, corresponding to rs7643677-C, was associated with increased *CMTM8* expression (35). In addition, it had been reported that rs6550109-T influenced *CMTM8* gene expression in monocytes, under both basal and LPS-activated conditions (*p*-value = 1.05 × 10^−13^ and *p*-value =1.03 × 10^-16^, respectively; **Supplementary Table S5**). Of note, rs6550109 is located into a distal enhancer-like signature classified by ENCODE (EH38E2189951), an element that could explain a regulatory function, but needs to be functionally validated.

To better define the cellular context in which the variant associated with post vaccination anti-S IgG levels acts, we tested whether our lead variant in the *CMTM8* locus colocalized with any genetic associations observed for 731 immune traits assessed in a sample of 3,757 individuals from the Sardinian population (33) (i.e. whether our trait and one of the immune traits shared one or more putative causal variants at the *CMTM8* locus).

We observed that the signal for anti-S IgG levels at the *CMTM8* locus colocalized with the signal affecting the expression of the adhesion molecule CD66b on PMN-MDSCs (**Figure 3B**) with a posterior probability for a shared causal variant (PP-H4) of 97.5% (**Supplementary Table S6**). To prioritize the potential shared causal variant between the two signals, we computed the probability for each variant in the region to be causal for both phenotypes, namely anti-S IgG levels and CD66b expression on MDSCs. We found that the lead variant for CD66b expression on MDSCs, rs12496184, had an 83% probability of affecting both traits, whereas rs7643677 had a 15% probability. Alleles of both lead variants, rs7643677 and rs12496184, had opposite effects on the two phenotypes, with an increase of anti-S IgG corresponding to a reduction of CD66b on PMN-MDSCs (**Figure 1**). PMN-MDSCs are a diversified cell population of myeloid origin, characterized by an immature phenotype and a strong capacity to suppress adaptive immune cell populations (18). Given that CD66b is a well-recognized granulocytic marker, we analyzed its expression within the granulocyte population using raw cytometry data from approximately 2,000 immunophenotyped samples from the SardiNIA Study cohort (33). Single locus genetic association analysis of the *CMTM8* gene with the newly assessed granulocyte data yielded no significant results (*p*-value=0.83), indicating that the *CMTM8* association is restricted to the PMN-MDSC cell type.

**Figure 4:**
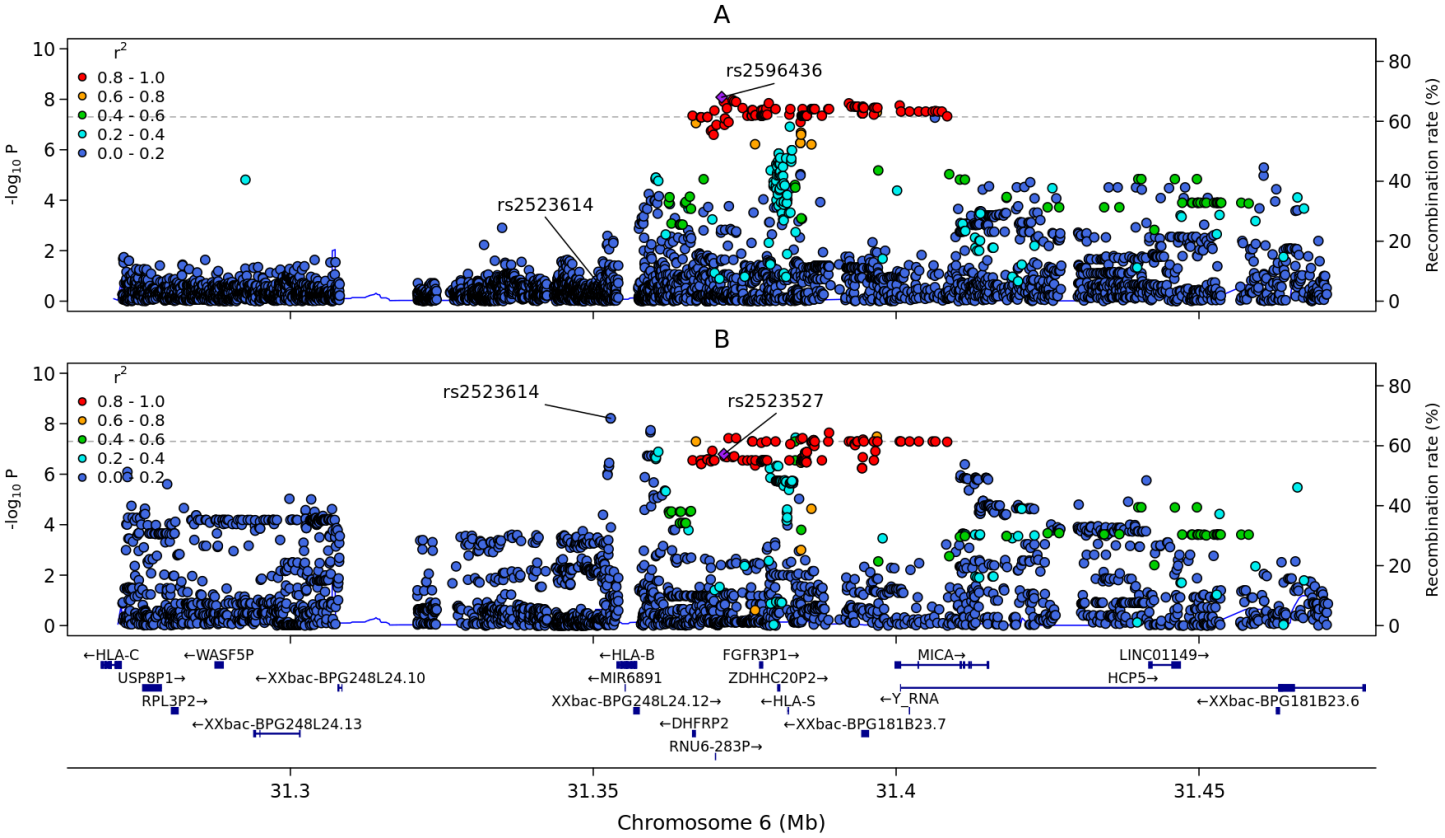
Regional association plots of the HLA region locus on chromosome 6 identified in the GWAS. Plots span a region encompassing ± 100 Kb from the lead variant rs2596436. Because rs2596436 is not present in the immunophenotype genetic map, its proxy rs2523527 (r^2^ = 1 in EUR), is reported. SNPs are plotted on the x-axis according to their position on chromosome 6, and *p*-values (−log_10_(p)) for their association with the traits are plotted on the y-axis. Variants are colored according to their LD (R^2^) with the lead SNP (purple diamond). **(A)** Regional plot from the GWAS on the anti-S IgG. **(B)** Regional plot for the immune trait (Naive DN (CD4-CD8-) %T cell).

In addition to the novel signal in the *CMTM8* gene, we confirmed the association of anti-S IgG levels with variants in the human leukocyte antigen (*HLA*) region (12), where the lead variant, rs2596436 (*p*-value = 7.709×10^-9^) mapped a few kb upstream of the *HLA-B* gene (**Figure 4A**). The lead variants reported in the same region by Esposito and colleagues (12) (rs2428479 and rs28366135) were in strong LD with rs2596436 (r^2^ = 0.98) and showed the same direction of effect (**Supplementary Table S7**). After conditional analyses in a +/- 100 kb region around rs2596436, we did not find other genome-wide significant signals. However, we found two additional suggestive independent associations (*p*-value<10^-6^), led by rs4472395 and rs111337511 (**Supplementary Table S8**). Notably, rs4472395 was in moderate LD with the top variant (rs1632893) from the same work (12) (r^2^ = 0.61).

We then searched for eQTLs within the *HLA* region associated with anti-S IgG levels (**Supplementary Table S9**). We found that rs2596436 was in strong LD (r^2^ = 0.98) with rs2596574, an eQTL for the *MICA* gene in brain (*p*-value= 5.26×10^-10^) (36). The rs2596574-A allele, corresponding to the rs2596436 top variant T allele, was associated with decreased *MICA* expression, while rs2596436-T was associated with increased anti-S IgG levels. In addition, rs2596436 was identified as an eQTL for *HLA-B, SKIV2L*, and *DDR1* in blood (*p*-value = 1.20 × 10 ^-31^, *p*-value = 1.13 × 10 ^-11^, and *p*-value = 2.32 ×10^-20^, respectively) (37).

The lead variant in the *HLA* region, rs2596436, was not present in the immunophenotype dataset of Orrù and colleagues (33). Therefore, we used rs2523527 as a proxy (r^2^ = 1 with rs2596436 in the EUR population). We then performed colocalization analysis in a +/- 100 kb region around rs2523527; the highest posterior probability of sharing a causal variant was observed with naïve double-negative (CD4neg CD8neg) T cells (PP-H4 = 89%) (**Figure 4B**; **Supplementary Table S6**). The rs2523527-G allele was associated with increased levels of circulating naïve double-negative T cells, as well as increased anti-S IgG production following BNT162b2 vaccination. Naïve double-negative T cells represent a minor population in T cells lineage with an unrecognized role in immune system. However, their expansion in inflammatory rheumatic disorders, including autoimmune lymphoproliferative syndrome, and systemic lupus erythematosus, leads to the hypothesis of a pro-inflammatory role (38).

## 4 Discussion

We conducted a GWAS to investigate the influence of host genetic factors on anti-S immunoglobulin G levels in response to SARS-CoV-2 vaccination in an Italian cohort of individuals without previous viral exposure. The large size of the original study cohort allowed us to apply stringent sample selection criteria, thereby including only individuals without serological evidence of prior infection who had received two doses of the BNT162b2 vaccine. This data filtering process, although reducing the overall sample size by 72.5% (from 7,169 to 1,968), effectively minimized heterogeneity and noise within the cohort, thereby enhancing our ability to detect association signals specific to the response to SARS-CoV-2 BNT162b2 vaccination.

The decrease in sample size was expected, as multiple types of COVID-19 vaccines were available across the Italian national territory. Indeed, 64% of the 128 million doses of COVID-19 vaccine administered in Italy consisted of BNT162b2 (Pfizer-BioNTech), while 24% were Spikevax (Moderna), 10% Vaxzevria (AstraZeneca), and 1% Jcovden (Janssen Johnson & Johnson). Furthermore, consistent with our data, only a subset of the population that had received two vaccine doses (80%) had been vaccinated with the same vaccine type for both.

Our GWAS on anti-S IgG values in the 1,968 selected samples identified a novel signal led by SNP rs7643677, with its C allele associated with reduced anti-S IgG levels following vaccination. rs7643677 is located in an intronic region of the *CMTM8* gene, which belongs to the chemokine-like factor gene superfamily (39) and is involved in tumor suppression and cell migration.

eQTL analysis of the lead variant rs7643677 supported its effect on *CMTM8* expression in both innate and adaptive immunity. Colocalization analysis with human immunophenotyping data further suggested an influence on CD66b expression in PMN-MDSCs. These cells represent one of the two major groups of MDSCs in humans and mice, classified according to their origin from the granulocytic myeloid cell lineage (40). Recently, their accumulation has been identified as a predictor of COVID-19 disease severity (21–23).

While most studies focus on cancers such as colon cancer, pancreatic cancer and gastric cancer (41, 42), little is known about the specific role of *CMTM8* on immune cells. A recent study showed that *CMTM8* was negatively correlated with CD8+ T cells, neutrophils, and dendritic cells, and suggested that *CMTM8* could be involved in promoting the process of epithelial-mesenchymal transition and forming the immunosuppression microenvironment through the TGF-β and the Notch pathway (43). Also, a suggestive involvement of the *CMTM8* gene has been observed in the context of the search for host genetic factors as determinants of HIV-1 acquisition (44). However, to our knowledge, there is no existing literature linking *CMTM8* gene variants specifically related to granulocyte function or MDSC expansion. Although these cells are commonly associated with pathological conditions, extensive evidence also supports a role for MDSCs in physiological, non-pathological settings such as vaccination, where they can limit vaccine efficacy (24). Indeed, the first study demonstrating the involvement of PMN-MDSCs during immunization was published in 2008, using a bivalent Salmonella vaccine (25). During SARS-CoV-2 infection or vaccination, early inflammatory activation likely drives PMN-MDSCs development primarily in peripheral tissues (24, 45). This hypothesis is supported by eQTL data which show that variant rs12496184 affects *CMTM8* expression in lung tissue (*p*-value=1.2×10^-5^) (37), the site where SARS-CoV-2 infection begins (46). Lung tissue also responds to the SARS-CoV-2 spike protein alone by triggering cell signaling cascades even in the absence of other viral components (46, 47).

Our study contributes to the understanding of the complex processes influencing vaccination efficacy by proposing a potential interplay among genetic, humoral, and cellular factors underlying the BNT162b2 vaccine response.

In detail, our hypotheses on the mechanisms underlying the genetic association of anti-S IgG levels with *CMTM8* can be summarized as follows: i) the signal led by rs7643677 affects the expression of the activation marker CD66b on PMN-MDSCs through a colocalizing variant, rs12496184, whose G allele is associated with increased CD66b expression on these cells, thereby enhancing their suppressive activity (CD66b is recognized as a granulocyte activation marker) (48–50); ii) as a consequence, in the presence of the rs12496184-G allele, B cells differentiate less effectively and produce lower levels of anti-S IgG; iii) in parallel, lower anti-S IgG production is observed in individuals carrying the rs7643677-C allele following vaccination with BNT162b2 (**Figure 1**).

It is worth noting that the *CMTM8* association appears to be restricted to the PMN-MDSCs cell type in our dataset. Given that CD66b is a well-recognized granulocytic marker, we initially hypothesized that the observed genetic association between *CMTM8* and CD66b expression on PMN-MDSCs might reflect a residual effect of a primary association with granulocytes. However, this was not supported by cytometric analysis of granulocytes on approximately 2,000 samples. Indeed, genetic association analysis between the *CMTM8* locus and the granulocyte trait yielded no significant results.

Interestingly, a nominally significant association was observed between rs7643677 and the trait “disease severity” in the COVID-19 Host Genetics Initiative study (14), with its C allele associated with a higher risk of developing severe symptoms or conditions that typically require hospitalization (effect size = 0.026, *p*-value=6.48×10^-3^). Consistently, the rs7643677-C was associated with lower levels of anti-S IgG in our data.

The influence of *CMTM8* within the myeloid compartment of the immune system is further supported by a recent study identifying it among the top 20 differentially expressed genes in influenza-stimulated monocytes (51).

Taken together, all these findings support a role for *CMTM8* in the regulation of the immune system, alongside its involvement in other biological pathways. The association of anti-S IgG levels following vaccination with *CMTM8* on chromosome 3 was not observed in previous studies with similar phenotypes, possibly because of differences in the quantification methods used for antibody detection. Some of the key features in our study that enabled us to obtain a highly informative phenotype were the strict filtering criteria, the relatively large sample size, the quantitative IgG detection method, and the single-center antibody assessment. We also confirmed a previously reported association involving the *HLA* region in the regulation of antibody response following COVID-19 vaccination (12). Notably, the lead SNP from our GWAS in this region regulates the expression of MHC class I molecules *HLA-B*, highlighting the pivotal role of this antigen presentation pathway in shaping the vaccine response. It also regulates the expression of genes influencing the immune response: in fact, rs2596436 is an eQTL for the *SKI2W* gene, which is involved in antiviral activity by blocking translation of poly(A) deficient mRNAs (52), for the *DDR1* gene, whose expression is associated with cytotoxic T cells and regulatory T cells function (53), and for the *MICA* gene, which exerts a crucial regulation on the immune system being the ligand for the NKG2D receptor that activates cell cytotoxic capabilities (54).

A key limitation of our study is its restriction to a relatively homogeneous Italian cohort. Although this homogeneity strengthens internal validity by reducing potential confounding due to population stratification, it may limit the external generalizability of our findings. Notably, population-genetic data indicate that the allele frequency of the lead SNP, rs7643677-C, is broadly comparable across major continental populations (44.9% in Europeans and 48.9% globally, based on 1000 Genomes Project reference data (55)). This concordance suggests that the functional relevance of this variant, and its potential contribution to immune-related pathways, may extend beyond European populations. Nonetheless, replication in ancestrally diverse cohorts remains necessary to establish the robustness and trans-ancestry transferability of the association.

Overall, our findings contribute to defining key aspects of vaccine response regulation, beyond the widely accepted role of the *HLA* region. Specifically, based on genetic evidence, we proposed a novel immunological mechanism that requires further support through functional and cellular studies. This mechanism operates in parallel with previously described processes involving both the adaptive immune system, such as antigen processing and presentation, and the innate immune system, including pathogen detection and response initiation via Toll-like receptors (TLRs) and RIG-I-like receptors (RLRs) (56–58).

## Data Availability

The GWAS summary statistics presented in the study are deposited in Figshare at the link: https://figshare.com/s/9adf6cac496d6274a78f. The names of the repository and accession numbers of the publicly available datasets used in this study can be found in the article/**Supplementary Material**. Further inquiries can be directed to the corresponding author.

## Group Members of the SerGenCovid19 Investigators

Giovanni Maga, Daniela Corda, Mario De Felice, Giorgio Iervasi, Alessandra Testa, Angela Celetti, Rosaria Catalano, Roberta Visconti, Vittoria D'Esposito, Laura Sabatino, Gabriele Trivellini, Stefano Turchi, Elisa Ferrari, Silvestro Conticello, Cristina Politi, Marco Mobrici, Giovanni Tripepi, Pizzini Patrizia, Rosa Maria Parlongo, Graziella D’Arrigo, Maria Carmela Versace, Antonella Lisa, Irene Cassaniti, Giuliana Esposito, Marcella Devoto, Edoardo Fiorillo, Anna Maristella Steri, Antonella Mulas, Alessandro Testori, Monia Lobina, Maria Grazia Piras, Fabio Cibella, Silvia Ruggieri, Gaspare Drago, Ilaria Cosentini, Teresa Colombo, Donald Baku, Dario Longo, Maurizio Martinelli, Lorenzo Luconi Trombacchi, Roberto Ravazzolo, Valentina Amenta, Franca Debole, Andrea Dell'Amico, Federico Volpini, Cinzia Caporale, Elena Mancini, Emanuela Midolo, Giuseppe Portella, and Pietro Formisano.

## Supplementary material

Supplementary Figure 1 Scatter plot of normalized IgG levels against the time elapsed in days between vaccine administration (second dose) and blood draw.

Supplementary Figure 2 Scatter plots of normalized IgG levels against age (A) and BMI (B). Box plots of normalized IgG levels against smoking status (C) and sex (D).
