# Supplementary figures and images for: CMTM8 variants influence BNT162b2 COVID-19 vaccination response by regulating granulocytic/polymorphonuclear myeloid-derived suppressor cell activity
Source: Front Immunol. 2026 Jan 23;17:1717058. doi: 10.3389/fimmu.2026.1717058 (PMC12875989; doi:10.3389/fimmu.2026.1717058)

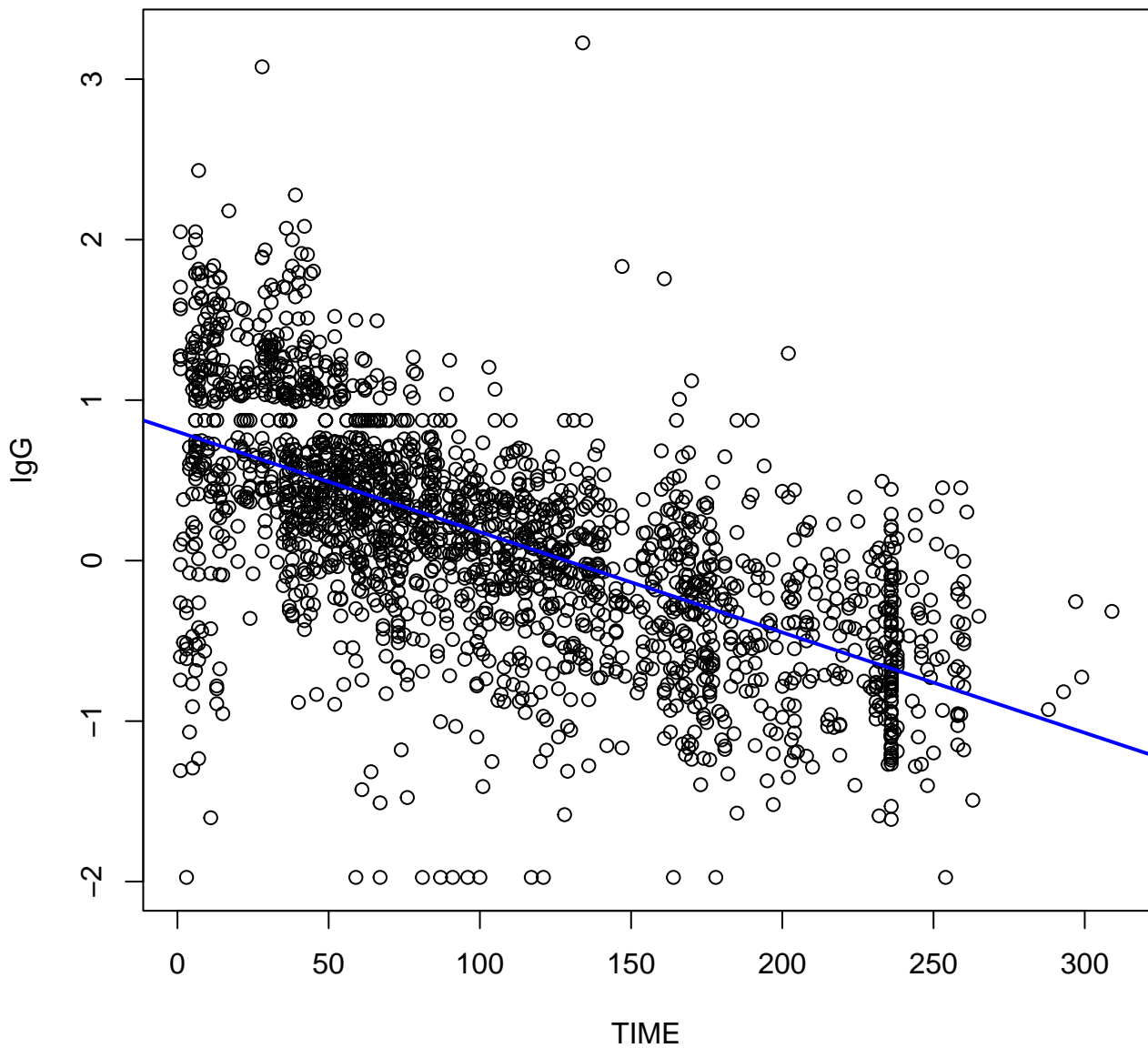

Supplement: Supplementary Figure 1 — Scatter plot of normalized IgG levels against the time elapsed in days between vaccine administration (second dose) and blood draw. [file DataSheet1.pdf]

**A**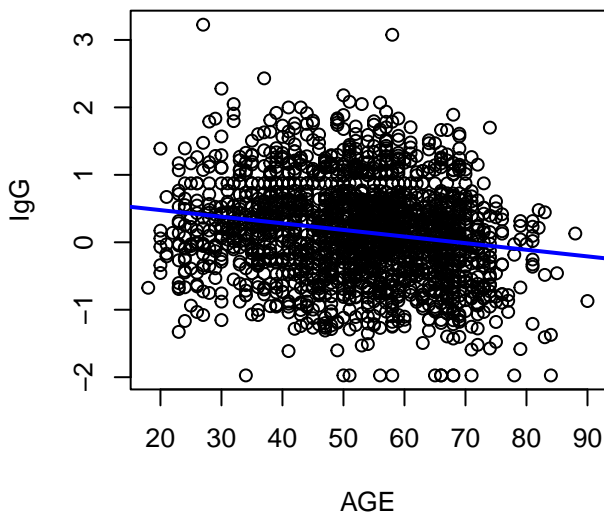**B**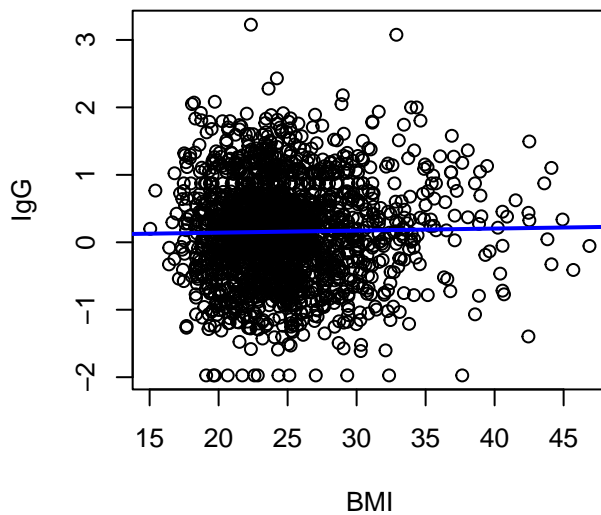**C**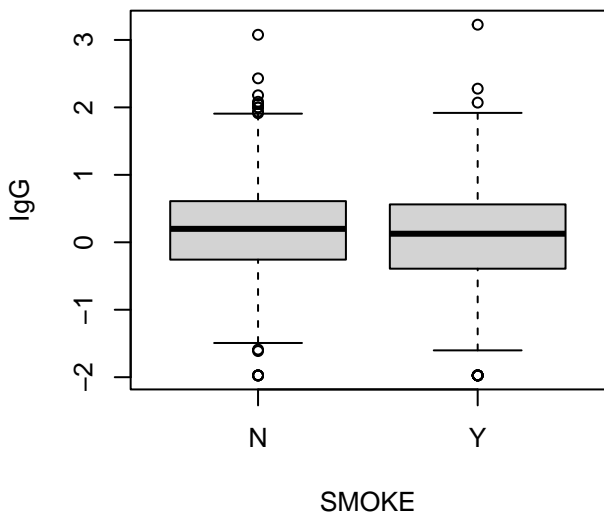**D**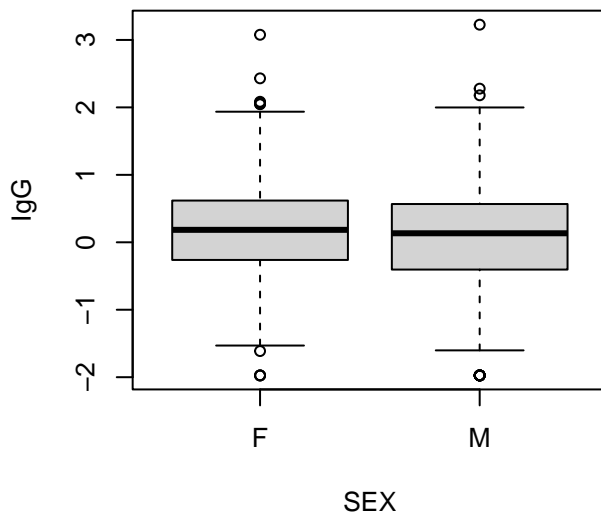

Supplement: Supplementary Figure 2 — Scatter plots of normalized IgG levels against age (A) and BMI (B). Box plots of normalized IgG levels against smoking status (C) and sex (D). [file DataSheet2.pdf]
