# Supplementary material for: CMTM8 variants influence BNT162b2 COVID-19 vaccination response by regulating granulocytic/polymorphonuclear myeloid-derived suppressor cell activity
Source: Front Immunol. 2026 Jan 23;17:1717058. doi: 10.3389/fimmu.2026.1717058 (PMC12875989; doi:10.3389/fimmu.2026.1717058)
Supplement: Supplementary file 3 [file DataSheet3.pdf]

SerGenCovid-19 Study Investigators: Giovanni Maga[6] , Daniela Corda[1], Mario De Felice[1], Giorgio Iervasi[3], Alessandra Testa[5], Angela Celetti[1], Rosaria Catalano[1], Roberta Visconti[1], Vittoria D'Esposito[1], Laura Sabatino[3], Gabriele Trivellini[3], Stefano Turchi[3], Elisa Ferrari[3], Silvestro Conticello[4], Cristina Politi[5], Marco Mobrici[5], Giovanni Tripepi[5], Pizzini Patrizia[5], Rosa Maria Parlongo[5], Graziella D'Arrigo[5], Maria Carmela Versace[5], Antonella Lisa[6], Irene Cassaniti[6], Giuliana Esposito[6], Marcella Devoto[7], Edoardo Fiorillo[7], Anna Maristella Steri[7], Antonella Mulas[7], Alessandro Testori[7], Monia Lobina[7], Maria Grazia Piras[7], Fabio Cibella[8], Silvia Ruggieri[8], Gaspare Drago[8], Ilaria Cosentini[8], Teresa Colombo[2], Donald Baku[2], Dario Longo[9], Maurizio Martinelli[10], Lorenzo Luconi Trombacchi[10], Roberto Ravazzolo[10], Valentina Amenta[10], Franca Debole[11], Andrea Dell'Amico[11], Federico Volpini[11], Cinzia Caporale[12], Elena Mancini[12], Emanuela Midolo[12], Giuseppe Portella[13], Pietro Formisano[13].

Dipartimento di Scienze Biomediche CNR:

1. *IEOMI (*già *IEOS),* I*stituto degli Endotipi in Oncologia, Metabolismo e Immunologia "G. Salvatore", Napoli*
2. *IBPM, Istituto di Biologia e Patologia Molecolari, Roma*
3. *IFC, Istituto di Fisiologia Clinica, Pisa,*
4. *IFC - Istituto di Fisiologia Clinica, URT di Firenze*
5. *IFC - Istituto di Fisiologia Clinica, Reggio Calabria*
6. *IGM, Istituto di Genetica Molecolare "Luigi Luca Cavalli-Sforza", Pavia*
7. *IRGB, Istituto di Ricerca Genetica e Biomedica, Cagliari*
8. *IRIB, Istituto per la Ricerca e l'Innovazione Biomedica, sede di Palermo*
9. IBB, *Istituto di biostrutture e bioimmagini,* Torino

10. *IIT, Istituto di informatica e telematica, Pisa*
11. *ISTI, Istituto di Scienza e Tecnologie dell'Informazione, Pisa*
12. *Centro Interdipartimentale per l'Etica e l'Integrità nella Ricerca, CNR.*
13. *Università Federico II, Napoli*
